# Meningococcal Meningitis Outbreaks in the African Meningitis Belt After Meningococcal Serogroup A Conjugate Vaccine Introduction, 2011–2017

**DOI:** 10.1093/infdis/jiz355

**Published:** 2019-10-31

**Authors:** Katya Fernandez, Clément Lingani, Olaolu Moses Aderinola, Kadadé Goumbi, Brice Bicaba, Zewdu Assefa Edea, Clément Glèlè, Badu Sarkodie, Agbeko Tamekloe, Armelle Ngomba, Mamoudou Djingarey, Ado Bwaka, William Perea, Olivier Ronveaux

**Affiliations:** 1 World Health Organization, Geneva, Switzerland; 2 World Health Organization, AFRO Intercountry Support Team for West Africa, Ouagadougou, Burkina Faso; 3 Nigeria Centre for Disease Control, Abuja, Nigeria; 4 Ministère de la Santé Publique du Niger, Niamey, Niger; 5 Ministère de la Santé, Ouagadougou, Burkina Faso; 6 Ethiopian Public Health Institute, Addis Ababa, Ethiopia; 7 Ministère de la Santé, Cotonou, Benin; 8 Ghana Health Service, Accra, Ghana; 9 Ministère de la Santé, Lomé, Togo; 10 Ministère de la Santé Publique du Cameroun, Yaoundé, Cameroon; 11 World Health Organization Regional Office for Africa, Brazzaville, Republic of the Congo

**Keywords:** public health surveillance, meningitis, bacterial, vaccination, Africa

## Abstract

**Background:**

In 2010–2017, meningococcal serogroup A conjugate vaccine (MACV) was introduced in 21 African meningitis belt countries. *Neisseria meningitidis* A epidemics have been eliminated here; however, non-A serogroup epidemics continue.

**Methods:**

We reviewed epidemiological and laboratory World Health Organization data after MACV introduction in 20 countries. Information from the International Coordinating Group documented reactive vaccination.

**Results:**

In 2011–2017, 17 outbreaks were reported (31 786 suspected cases from 8 countries, 1–6 outbreaks/year). Outbreaks were of 18–14 542 cases in 113 districts (median 3 districts/outbreak). The most affected countries were Nigeria (17 375 cases) and Niger (9343 cases). Cumulative average attack rates per outbreak were 37–203 cases/100 000 population (median 112). Serogroup C accounted for 11 outbreaks and W for 6. The median proportion of laboratory confirmed cases was 20%. Reactive vaccination was conducted during 14 outbreaks (5.7 million people vaccinated, median response time 36 days).

**Conclusion:**

Outbreaks due to non-A serogroup meningococci continue to be a significant burden in this region. Until an affordable multivalent conjugate vaccine becomes available, the need for timely reactive vaccination and an emergency vaccine stockpile remains high. Countries must continue to strengthen detection, confirmation, and timeliness of outbreak control measures.

The African meningitis belt, extending across sub-Saharan Africa from Senegal to Ethiopia, is known for meningitis seasonal hyperendemicity as well as periodic large-scale outbreaks [1]. Before 2010, *Neisseria meningitidis* serotype A (NmA) was responsible for most epidemics, while other serogroups (W and X) also caused epidemics [2, 3]. Reactive vaccination of affected populations with serogroup-specific vaccines is an essential component of the meningitis outbreak control strategy [3]. The development of a meningococcal serogroup A conjugate vaccine (MenAfriVac, MACV) for Africa enabled the integration of preventive vaccination into the control strategy. Since the progressive introduction of MACV in 21 meningitis belt countries (2010–2017), NmA cases have been reduced dramatically and epidemics have been successfully eliminated from the region [4, 5]. A study conducted in 9 countries that carried out mass campaigns with MACV (Benin, Burkina Faso, Chad, Côte d’Ivoire, Ghana, Mali, Niger, Nigeria, and Togo) found a 58% decline in incidence of suspected meningitis, a >99% decline in incidence of confirmed NmA meningitis, a 59% decline in risk of epidemics, and an increase in incidence of non-NmA meningitis (incidence rate ratio 2.76; confidence interval, 1.21–6.30) [6].

Outbreaks due to non-NmA serogroups are still reported in the region, necessitating continuing outbreak response measures, particularly reactive vaccination [5]. Since its establishment in 1997, the International Coordinating Group (ICG) on vaccine provision for epidemic meningitis control has managed an emergency vaccine stockpile to sustain country access to emergency vaccines [7]. However, the provision of vaccine covering non-NmA serogroups is increasingly limited, as manufacturers have started phasing out the production of polysaccharide vaccines, the main vaccines used for outbreak response in Africa [5, 8], and privileging the production of the more effective but costly conjugate vaccines.

We present here a description and analysis of *N. meningitidis* epidemics that have occurred after MACV introduction through 2017, including an analysis of the reactive vaccination.

## MATERIAL AND METHODS

### Definitions

World Health Organization (WHO) definitions for cases and outbreaks were used in this analysis, as shown in Table 1. The epidemic season was defined as the period between weeks 1 and 26, from 1 January of each year.

**Table 1.** Case and Outbreak Definitions

Category	Definition
Suspected meningitis case	Any person with sudden onset of fever (>38.5°C rectal or 38.0°C axillary) and 1 of the following signs: neck stiffness, flaccid neck (infants), bulging fontanelle (infants), convulsion or other meningeal signs [9, 10].
Confirmed meningitis case	Any person with meningeal signs and identification of a causal pathogen (*Neisseria meningitidis*, *Streptococcus pneumoniae*, *Haemophilus influenzae* type b) in the cerebrospinal fluid by culture, polymerase chain reaction, or rapid diagnostic test [9, 10].
Alert threshold	An attack rate of 3 suspected cases per 100 000 inhabitants per week in a district or subdistrict with populations >30 000 or, in populations <30 000, 2 cases in 1 week or a higher incidence than in a nonepidemic year [9]. Crossing this threshold triggers the reinforcement of surveillance.
Epidemic threshold	An attack rate of 10 suspected cases/100 000 inhabitants in 1 week in a district or subdistrict with populations >30 000 or, in populations <30 000, 5 cases in 1 week or a doubling of incidence in a 3-week period. In special situations with high transmission risk (eg, mass gatherings, refugee camps, displaced persons, or closed institutions), 2 confirmed cases within 1 week are considered an epidemic [9].
Outbreak	Defined operationally as a situation in which the epidemic threshold has been crossed, triggering response actions: the launch of vaccination campaigns and the use of a specific antibiotic treatment protocol.

### Data Collection and Analysis

The WHO enhanced meningitis surveillance (ES) network was established across the African meningitis belt in 2003, initially in 8 countries [2]. Standard methods were developed to detect and notify cases, including standard operating procedures, standard case definitions, intervention thresholds (see Table 1), laboratory standards, and data collection tools. From 2011 to 2017, the number of countries participating in the ES network increased from 13 to 24 [11].We reviewed data reported to the WHO ES regional network after MACV introduction in the 21 countries (Benin, Burkina Faso, Cameroon, Chad, Central African Republic, Côte d’Ivoire, DRC, Ethiopia, Gambia, Ghana, Guinea, Guinea-Bissau, Mali, Mauritania, Niger, Nigeria, Senegal, South Sudan, Sudan, Togo and Uganda) that had completed mass campaigns. We excluded data from the Democratic Republic of Congo as the majority of the country is considered to be outside the meningitis belt and therefore the intervention thresholds are not considered applicable [5, 11]. National-level data included numbers of suspected cases and deaths, laboratory confirmation (by polymerase chain reaction, culture, and agglutination) of cases, and number of epidemic districts. Case fatality ratios (CFR) were calculated using all the reported suspected cases as denominator. The data used to describe the outbreaks included the aggregate, weekly, district-level data from the ES regional database, as well as individual outbreak reports and applications for vaccines from the ICG emergency vaccine stockpile. We also used anonymized line list data, which included more detailed district-level laboratory confirmation data when this was available, as was the case in Ghana 2016, Niger 2015–2017, and Nigeria 2017. To calculate attack rates per 100 000, we used the official district-level population sizes as reported by each country to WHO, which are updated annually.

 Districts reaching the epidemic threshold in 1 country were grouped as a single outbreak when time and place converged. An outbreak was attributed to 1 serogroup, defined as the serogroup reported in >50% of confirmed cases. When the proportion of another serogroup was sizable (≥30%) this was indicated. We did not consider pneumococcal meningitis outbreaks in this analysis. Molecular analyses, conducted by the WHO Collaborating Centers for Meningitis, on a limited number of cerebrospinal fluid samples/isolates, allowed genotypic characterization of outbreak strains.

Vaccination data (administrative coverage, response timelines, and number of vaccinated individuals) were obtained from the ICG vaccine request database and vaccination reports. Reporting of epidemic areas was done at district level, as per the ES data reporting. However, reactive vaccination sometimes targeted populations at the subdistrict level, resulting in partial vaccination of districts.

We estimated the timeliness of response, defined as the time between the date of crossing the epidemic threshold (last day of the epidemiological week when the threshold was crossed) and the date when the reactive vaccination campaign was started. We further characterized this timeline as: (1) the delay between crossing of the epidemic threshold and the date when the ICG vaccine request was approved (as a proxy for the time needed to properly document an outbreak and decide on response); and (2) vaccination delay between vaccine request approval and the start of vaccination (ie, the time taken for vaccine deployment and campaign preparation).

## RESULTS

### Epidemiological Description

Between 2011 and 2017, a total of 17 outbreaks were reported from countries that had introduced MACV. These occurred in 8 countries and affected 113 districts, for a total number of 31 786 suspected cases (yearly range from 129 in 2011 to 16 547 in 2017; Table 2). From 2011 to 2014, only 1 outbreak was reported per year, while from 2015 to 2017, 3 to 6 outbreaks per year were reported. All of them were reported during the epidemic season except for 1 outbreak in a refugee camp in Ethiopia (weeks 40 to 50). The most affected country was Nigeria, which reported 5 outbreaks with a total of 17 375 suspected cases and 55 epidemic districts, followed by Niger, which reported 4 outbreaks (9343 cases, 21 epidemic districts). Burkina Faso was affected by a large outbreak in 2012 (2372 cases, 13 epidemic districts) but did not report outbreaks subsequently. Togo was affected by 1 large outbreak in 2016 [12] and 2 in 2017 (1812 cases and 11 epidemic districts in total). Benin, Cameroun, Ethiopia, and Ghana reported 1 outbreak each during this period. Outbreaks ranged in size from 18 cases (refugee camp outbreak, Ethiopia 2015) to 14 542 cases (Nigeria, 2017), with a median of 467 suspected cases, and affected between 1 and 37 districts (median 3). Two outbreaks were considered as special situations, where 2 confirmed cases in a week constituted an epidemic: 1 was reported in an Ethiopian refugee camp in 2015 and the other in a Cameroonian prison in 2017. The number of cases, 18 and 25 respectively, were the lowest among all outbreaks, with CFR of 0% and 32%. Average CFR for all outbreaks, excluding these 2 special situations, was 6% (range 2%–11%). The number of cases reported during the 17 outbreaks represented 45% of the total number of cases (week 1 to 26) reported after MACV introduction, ranging from 3% in 2011 to 83% in 2017.

**Table 2.** Description of Meningococcal Meningitis Outbreaks in the Meningitis Belt, 2011–2017

Year	Country	Number of Epidemic Districts	Predominant Pathogen	Clonal Complex	Number of Cases per Outbreak	Number of Confirmed Cases per Outbreak	Proportion of Confirmed Outbreak Cases (%)	Number of Deaths per Outbreak	Case Fatality Ratio (%)	Cumulative AR, Average (Range)	Number of Years After MACV Mass Campaigns
2011	Niger	1	NmW	ST-11	129	66	51	13	10	43 (NA)	1
2012	Burkina	13	NmW	ST-11	2372	532	22	219	9	85 (59–111)	2
2013	Nigeria	3	NmC	ST-10217	329	7	2	13	4	67 (46–87)	1
2014	Nigeria	6	NmC	ST-10217	467	10	2	50	11	100 (12–261)	2
2015	Ethiopia	1	NmC	ST-103	18	6	33	0	0	NA	2
2015	Niger	13	NmC	ST-10217	6775	1456	21	489	7	203 (27–992)	5
2015	Nigeria	8	NmC	ST-10217	1854	20	1	80	4	186 (29–1037)	3
2016	Ghana	10	NmW	NAV	763	138	18	49	6	97 (31–195)	4
2016	Niger	3	NmC	ST-10217	760	150	20	23	3	83 (39–116)	6
2016	Nigeria	1	NmC	ST-10217	183	35	19	7	4	183 NA	4
2016	Togo	9	NmW	ST-11	1589	346	22	83	5	142 (70–233)	1
2017	Benin	1	NmC	NAV	78	11	14	6	8	189 NA	5
2017	Cameroun	1	NmC	ST-175	25	5	20	8	32	NA	5
2017	Niger	4	NmC	ST-10217	1679	428	25	76	5	112 (62–198)	7
2017	Nigeria	37	NmC	ST-10217	14 542	563	4	1163	8	131 (27–666)	5
2017	Togo	1	NmW	ST-11	100	22	22	5	5	138 (NA)	2
2017	Togo	1	NmW	ST-11	123	34	28	3	2	37 (NA)	2
Total		113			31 786	3815	20^a^	2287	6^b^	119.7	

Abbreviations: AR, attack rate; NA, not applicable; NAV, not available; NmA, NmC, NmW, and NmY, *Neisseria meningitidis* serogroups.

^a^Median.

^b^Average excluding Cameroon and Ethiopia (special situations outbreaks).

NmC was identified as the predominant pathogen in 11 of the 17 outbreaks (Benin, Cameroun, Ethiopia, Niger, and Nigeria), where a total of 78 epidemic districts and 26 710 cases were reported. NmW was the predominant pathogen in 6 outbreaks (Niger 2011, Burkina Faso, Ghana, and Togo) in a total of 35 districts and with 4935 cases. Notably, there were no NmA outbreaks reported. NmX was not predominant in any of the outbreaks, but its proportion was sizable in 1 outbreak, in Togo in 2017, where it represented 37% of the *N. meningitidis* cases identified.

**Figure 1.**
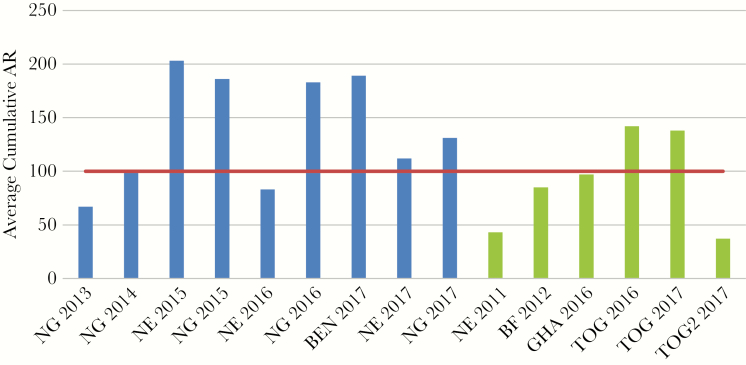
Cumulative attack rates per outbreak (district average), stratified by serogroup, excluding special situations outbreaks in Cameroon and Ethiopia. *Neisseria meningitidis* serogroup C outbreaks are shown in blue and *N. meningitidis* W outbreaks in green. Red line indicates epidemic criterion. Abbreviations: AR, attack rate; BEN, Benin; BF, Burkina Faso; GHA, Ghana; NE, Niger; NG, Nigeria; TOG, Togo.

The average district cumulative attack rate per outbreak was 120 cases/100 000, ranging from 37/100 000 population (Togo 2017, 1 district) to 203/100 000 (Niger 2015, 13 districts). Cumulative attack rates per district were as high as 1037 cases/100 000 population (Aliero, Nigeria, 2015). The average district cumulative attack rate was higher for NmC outbreaks (139 cases/100 000 population/year, 9 outbreaks, 76 epidemic districts) than for NmW outbreaks (90 cases/100 000 population/year, 6 outbreaks, 35 epidemic districts; Figure 1). The 2 special situation outbreaks were excluded from this comparison.

The median proportion of suspected cases that were laboratory confirmed per outbreak was 20% (range 1% Nigeria 2015 to 51% Niger 2011). For the 3 largest outbreaks (>2000 cases: Nigeria 2017, Niger 2015, and Burkina 2012) these proportions were 4%, 17%, and 22%, respectively.

### Molecular Analyses

Molecular analyses of outbreak strains led to the identification of the emergence of a highly epidemic NmC strain, sequence type (ST)-10217. This strain was responsible for the large-scale NmC outbreaks in Niger and Nigeria [13]. The small NmC outbreaks in Ethiopia and Cameroun were caused by 2 different STs (ST-11592 and ST-2881, respectively) belonging to clonal complexes unrelated to ST-10217 (Table 2). The NmW strains responsible for the epidemics during the study period belonged to clonal complex ST-11, which has been circulating in the meningitis belt since 2000 [14]. No molecular information was available for the Benin and Ghana outbreaks.

### Epidemic Response

A reactive vaccination campaign was organized to respond to 14 (78%) of the outbreaks and in 74% of the epidemic districts (84/113), as well as in 30 other affected districts (subdistrict vaccination), with a total of 5 724 603 people being vaccinated. The administrative vaccination coverage rate achieved ranged from 66% to 107% (median 87%; Table 3). Vaccination was conducted in a median of 5.5 districts per outbreak (range 1 to 35) and 408 900 people were vaccinated per outbreak (range 6720 in Cameroun in 2017 to 2 008 400 in Nigeria in 2017). In all outbreaks where reactive vaccination was conducted, a polysaccharide (ACW or ACYW) vaccine was used, covering 4.7 million people. For 2 of the outbreaks, a conjugate vaccine was used in 10 of the affected districts (ACYW conjugate vaccine in 1 of 15 districts vaccinated in Niger 2015 and monovalent C conjugate in 9 of 35 districts vaccinated in Nigeria 2017), covering 982 150 people (Table 3). As this was the first time these vaccines had been used for outbreak response in Africa, and to maximize potential impact (eg, herd protection) and for monitoring purposes, conjugate vaccines were used under specific conditions (vaccination of whole districts, distribution of vaccination cards, coverage survey, and strengthened monitoring).

**Table 3.** Reactive Vaccination Campaign Uptake and Vaccine Used During 2011–2017 Meningococcal Outbreaks

Year	Country	Number of Districts Vaccinated	Type of Vaccine Used	Vaccination Coverage (%)	Number of People Vaccinated
2011	Niger	1	PS ACW	75	94 286
2012	Burkina	3	PS ACYW	107	284 218
2015	Ethiopia	2	PS ACW	83	90 251
2015	Niger	15	PS ACW, PS ACYW, Conj ACYW	74	1 200 352
2015	Nigeria	17	PS ACYW	66	340 538
2016	Ghana	3	PS ACW	100	139 700
2016	Togo	9	PS ACYW	99	553 288
2016	Niger	8	PS AC	83	206 755
2016	Nigeria	9	PS AC	87	171 492
2017	Nigeria	35	PS ACYW; PS AC; Conj C	86	2 008 400
2017	Niger	9	PS AC	86	470 837
2017	Togo	1	PS ACYW	99	50 559
2017	Cameroun	1	PS ACYW	93	6720
2017	Togo	1	PS ACW	99	107 207
Total		114			5 724 603

Abbreviations: A, C, W, and Y, *Neisseria meningitidis* serogroups; Conj, conjugate; PS, polysaccharide.

The time lag to start the vaccination ranged from 10 to 64 days (median 36 days) (Table 4). In 6 out of 13 of the outbreaks the response delay was within 4 weeks of crossing the epidemic threshold. The investigation delay ranged from 5 to 33 days and was estimated at 51% of the total delay, while the vaccination delay ranged from 10 to 31 days (49% of the delay).

**Table 4.** Timeliness of Reactive Vaccination

Year	Country	No. of Districts Vaccinated	Investigation Delay, Average, Days^a^	Vaccination Delay, Average, Days^b^	Response Delay, Average, days^c^
2011	Niger	1	30	NA	10
2012	Burkina Faso	3	NA	24	NA
2015	Ethiopia	2	33	31	64
2015	Niger	12	15	13	28
2015	Nigeria	8	16	23	38
2016	Ghana	3	22	14	36
2016	Togo	9	8	19	27
2016	Niger	3	28	25	53
2016	Nigeria	7	33	NA	25
2017	Nigeria	28	28	19	46
2017	Niger	4	22	15	38
2017	Togo	1	18	20	38
2017	Cameroun	1	10	10	20
2017	Togo	1	5	20	25
Total No. districts and median delays		83	22.0	19.5	36.0

For Burkina Faso, investigation and response delays were not applicable as the districts vaccinated were not included in the initial request and for the response delay. Vaccination delay was not applicable (for the 2 cases ) when vaccination in the targeted districs was initiated with national stockpiles before arrival of vaccines from ICG.

Abbreviation: NA, not applicable.

^a^Number of days between epidemic threshold crossing and International Coordinating Group (ICG) request approval.

^b^Number of days between ICG request approval and start of vaccination.

^c^Number of days between epidemic threshold crossing and start of vaccination.

## DISCUSSION

Our review shows that meningococcal outbreaks of large magnitude continue to occur in the countries of the African meningitis belt after introduction of MACV. While NmA outbreaks have disappeared, and the overall risk of epidemics has substantially declined [6, 15], non-NmA outbreaks continue to affect the region, frequently with high attack rates. Ten of the 15 outbreaks (excluding the 2 outbreaks in special situations) described in this analysis met the 100 cases/100 000 population seasonal cumulative incidence, which has been previously used to define meningitis belt epidemics [16, 17]. Seven of the 10 higher incidence outbreaks were caused by the new NmC strain, ST-10217. This strain caused large-scale outbreaks affecting more districts per outbreak and higher incidence rates than the NmW outbreaks during the same period. Yearly incidence reached 1000 cases/100 000 population in 2 districts in Niger and Nigeria, a level comparable to the highest incidence observed in the large-scale NmA outbreak in Nigeria in 2009 (728 cases /100 000 population/year) [18]. Nonetheless, NmW also caused large-scale outbreaks with high incidence rates (15 districts with yearly incidence ≥100 cases /100 000 population). NmX was detected in only 1 outbreak, but it has caused major outbreaks in the past [19, 20], and was detected increasingly in 2017 and 2018 [21, 22]. This requires close monitoring, especially given the lack of a vaccine to cover this serogroup.

Average CFRs in our review were within the range of those found in NmA epidemics in the region, which oscillated around 10% [23] and were as low as 4% in the Nigeria 2009 outbreak [24]. It should be noted that ascertainment of meningitis deaths, and therefore estimation of CFR, is often variable and dependent on a country’s surveillance system and performance [25].

These results also point to the largely unpredictable behavior of *N. meningitidis* outbreaks [26]. In 2018, a considerable decrease in epidemic activity was reported compared to 2017. NmC continued to cause outbreaks in Nigeria, but only in 8 districts, as well as at subdistrict level in Niger [27]. This decrease in epidemic activity parallels that observed in 2016, following the large-scale NmC epidemic of 2015.

Our results call for continued and renewed investment in effective outbreak response. Reported reactive campaign coverage rates were globally high. However, in 3 outbreaks they were below 80%, highlighting remaining difficulties to reach populations in certain areas. Furthermore, these figures only reflect reported administrative coverage rates, as coverage surveys in reactive campaigns could not be systematically conducted. Ensuring the timeliness of reactive vaccination remains the biggest challenge for effective outbreak response. Reactive vaccination should be conducted within 4 weeks of crossing the epidemic threshold to maximize its impact [17, 28]. This was achieved in only 46% of the outbreaks where a response was organized. Response delays were both due to challenges in documenting and confirming the outbreak, as well as due to challenges to rapidly deploy the vaccines and organize the vaccination campaigns. Gaps in laboratory confirmation in outbreak settings are shown in this analysis. Limited and late laboratory confirmation has been highlighted as a key bottleneck in the submission of ICG vaccine requests and decision making on vaccine release [29]. Delays in vaccine deployment and campaign planning also hampered the response. Efforts to maintain adequate vaccine stockpiles and to optimize international vaccine delivery procedures should be pursued to alleviate the delays. Limited emergency stockpiles undermined the timeliness and effectiveness of outbreak response in 2012 and 2015 [30, 31] and remain a serious concern. In 2017–2018, WHO and partners called for concerted action to increase global vaccine availability in the short and long term in the face of the expansion of NmC in the region and continued low vaccine stockpile levels [32].

While the ICG emergency stockpile is mostly composed of polysaccharide vaccines, almost 1 million doses of conjugate vaccines were deployed to respond to large NmC outbreaks. This is the first time conjugate vaccines have been used for a non-A serogroup meningitis outbreak response in Africa. In contrast to polysaccharide vaccines, which are only recommended for reactive vaccination, conjugate vaccines can confer herd protection and protect infants, making them a superior control tool and extending their benefit beyond outbreak control to outbreak prevention. Given these characteristics, stockpiling conjugate vaccines should be favored despite their higher prices, as their use could be optimized by repurposing for vaccination of high-risk areas.

The epidemiological description and analyses presented in this review are mainly derived from ES data, analyzed and disseminated weekly during the meningitis season through a bulletin produced by WHO [11]. The population-based ES, currently covering all districts from 24 countries in the region, produces reliable, essential data for decision making during outbreak response [2]. Maintenance and strengthening of ES in all countries of the belt is recommended, accompanied by strong laboratory capacity [25].

ES implementation can be improved, however: a few African meningitis belt countries have yet to join the ES network and some report data irregularly and incompletely, particularly laboratory results. These limitations also affected our analysis but we could not assess the completeness of ES data.

The different sensitivities and specificities of the ES country systems were described in the 2015 analysis of this network [2]. The epidemic threshold used to detect outbreaks is based on the number of suspected cases, identified according to standard case definitions. However, ensuring the correct application of the case definition requires constant health worker training, a challenge in many countries. As the proportion of confirmed cases was only 20% and given the changing epidemiological context after MACV rollout, we cannot ascertain whether the remaining proportion of suspected cases are true bacterial meningitis cases. Nevertheless, this proportion of laboratory confirmation was considered informative enough, as per WHO standard operating procedures [9, 10], to guide outbreak response decision making.

The epidemic threshold is defined for populations below 100 000, but it is commonly applied and reported at district level, with populations often >200 000 [17]. Furthermore, district population estimates reported by countries may be underestimated, as evidenced by the vaccination coverage rates ≥100% reported in some countries. Outbreaks at subdistrict level and in hard-to-reach areas may therefore have been missed, as well as small outbreaks not meeting the standard regional definition. This was likely the case of a reported outbreak in Mali, in 2017 [33], which was not included here as it did not cross the epidemic threshold as per ES data. Of note, the definition of outbreaks in the meningitis belt is derived from historical NmA epidemics and calculation of weekly attack rates at the district level. The applicability of this definition and outbreak thresholds was reviewed in 2014 for NmW outbreaks and was found to be adequate, except for the alert threshold, which was lowered [17, 28]. This was confirmed for NmC outbreaks in 2015 [34], and was recently reevaluated and found applicable [26].

We report on 2 special situation outbreaks, which can be considered as outliers among the list of outbreaks described. This is in consideration of both the low number of cases reported as well as to the seasonality, which is irrespective of the meningitis season. These outbreaks are defined by a particular context of high transmission risk, which is not due to the climatic conditions (seasonality) but rather to crowding. Their inclusion in this review is justified as they require special surveillance and control measures.

Our analysis confirms previous observations on the expansion and high epidemic potential of the NmC ST-10217 strain [34]. Furthermore, molecular characterization analysis, undertaken by the WHO Collaborating Centers for Meningitis of 2011–2016 meningococcal strains in the belt, found ST-10217 to be the second most predominant strain (after ST-11), despite it being collected only from 2013 onwards [14]. The emergence of this hyperinvasive NmC strain highlights the need for reinforced surveillance, particularly the laboratory and molecular components, which remain weak in Africa. Initiatives to strengthen case-based surveillance, reinforce laboratory capacity, and enable systematic molecular characterization of epidemic strains [15, 35] should be encouraged and extended.

In conclusion, despite the remarkable success of MACV introduction, *N. meningitidis* epidemics due to non-A serogroups still represent a significant burden in the region but remain largely unpredictable. Country preparedness together with timely outbreak detection and response are therefore indispensable to reduce the morbidity and mortality associated with meningococcal epidemics. At the global level, maintaining country access to an adequate supply of vaccine for response remains a high priority, until affordable multivalent (including the X serogroup) conjugate vaccines are made readily available and introduced in all at-risk countries, and particularly those of the meningitis belt [36].

## Notes

Presented in part: International Pathogenic Neisseria Conference, Pacific Grove, California, 23–28 September 2018.

## Notes

***Financial support***. This work was funded by the MenAfriNet consortium (www.menafrinet.org) through a grant from the Bill & Melinda Gates Foundation (OPP1084298).

***Potential conflicts of interest***. All authors: No reported conflicts of interest. All authors have submitted the ICMJE Form for Disclosure of Potential Conflicts of Interest. Conflicts that the editors consider relevant to the content of the manuscript have been disclosed.
